# Iridium wire grid polarizer fabricated using atomic layer deposition

**DOI:** 10.1186/1556-276X-6-558

**Published:** 2011-10-25

**Authors:** Thomas Weber, Thomas Käsebier, Adriana Szeghalmi, Mato Knez, Ernst-Bernhard Kley, Andreas Tünnermann

**Affiliations:** 1 Friedrich-Schiller-University Jena, Institute of Applied Physics, Max-Wien-Platz 1, 07749 Jena, Germany; 2 Max-Planck-Institute of Microstructure Physics, Weinberg 2, 06120 Halle, Germany; 3 Fraunhofer Institute for Applied Optics and Precision Engineering, Albert-Einstein-Straße 7, 07745 Jena, Germany

**Keywords:** optics, nanostructure fabrication, polarizing devices

## Abstract

In this work, an effective multistep process toward fabrication of an iridium wire grid polarizer for UV applications involving a frequency doubling process based on ultrafast electron beam lithography and atomic layer deposition is presented. The choice of iridium as grating material is based on its good optical properties and a superior oxidation resistance. Furthermore, atomic layer deposition of iridium allows a precise adjustment of the structural parameters of the grating much better than other deposition techniques like sputtering for example. At the target wavelength of 250 nm, a transmission of about 45% and an extinction ratio of 87 are achieved.

## 1 Introduction

Wire grid polarizers offer a large spectral working range, small feature size, and good integrability and are of utmost importance for various applications such as microscopy or imaging systems. Generally, a wire grid polarizer consists of a periodical arrangement of conductive (metallic) wires on a transparent substrate. Upon illumination, a wire grid polarizer shows a higher transmission for light with the electrical field vector perpendicular to the wires (transverse magnetic (TM) polarization) than for the parallel counterpart (transverse electric (TE) polarization). In addition to the transmission of TM-polarized light, the extinction ratio which is defined by the ratio of TM- and TE-polarized light is another characteristic optical property of a wire grid polarizer. The spectral working range and the optical properties of a wire grid polarizer are determined by the grating material and the structural parameters of the grating such as period, grating height, or ridge width. First wire grid polarizers for the IR spectral region were demonstrated by Bird and Parrish [1] in 1960. Subsequent work in the past years showed polarizers for the terahertz [2], IR [3], visible [4], and UV [5] spectral ranges. Decreasing the operation wavelength requires smaller grating periods. For a wire grid polarizer, it is necessary that only the zeroth diffraction order is propagating. For this purpose it is known that the grating period must be much smaller than the incident wavelength. Consequently for a target wavelength of 250 nm a period of about 100 nm is required. The fabrication of such a high frequency metallic grating with high aspect ratios is technologically very challenging and demands a sophisticated lithography and metal structuring process. Possible lithographical processes include nanoimprint lithography [5], interference lithography [6], or electron beam lithography [7]. Metal structuring can be accomplished by means of a lift off process [8], dry etching [9], or a spatial frequency doubling technique [10].

In this Letter, we present an iridium wire grid polarizer for broadband applications down to a wavelength of 250 nm fabricated by a spatial frequency doubling technique based on ultrafast electron beam lithography and atomic layer deposition (ALD). Moreover, we compare the suitability of ALD and sputtering as deposition technique for the frequency doubling process. In our previous work, we already demonstrated that iridium is suitable as an alternative grating material to the frequently used aluminum [11]. The refractive index of iridium and aluminum is shown in [12]. Compared with aluminum, iridium shows a superior corrosion resistance and the optical properties of an iridium wire grid polarizer generally comply with the requirements for broadband applications with wavelength of less than 300 nm. To realize these aimed optical properties, a grating with a period of 100 nm, a grating height of about 150 nm, and a ridge width of approximately 35 nm was prepared. ALD was the method-of-choice for the frequency doubling process [13] as it is superior in terms of step coverage and uniformity of the coating of high aspect ratio structures [14,15] compared to conventional sputtering techniques, especially since a straight forward ALD process for iridium is available [16,17]. It provides the possibility to accurately adjust the ridge width of the metal grating simply by controlling of the metal layer thickness through the number of ALD cycles. Furthermore, atomic layer deposition is a non-line-of-sight deposition technique and thus not affected by shadowing effects as it is observed in physical deposition processes under oblique incidence. Hence, the value of the ridge width of the fabricated iridium grating is not limited compared to our previous work [11] where sputtering was used as deposition technique.

**Figure 1:**
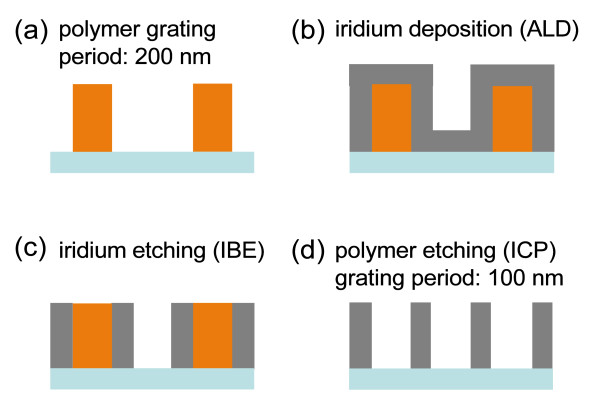
**Schematic illustration of the frequency doubling process for the fabrication of a wire grid polarizer**.

The fabrication of the high-frequency iridium grating was realized using a spatial frequency doubling process. Initially, a grating with twice the period of the final grating structure, in this case 200 nm, is fabricated. The requirement imposed to this grating is to permit selective removal against iridium and the substrate material. A tempered photoresist is chosen as material because of its good processibility. The photoresist was spin coated onto a fused silica substrate and tempered afterward. Then, the photoresist layer was covered with a chromium layer and an electron beam-sensitive resist (ZEP 520). The 200 nm period resist pattern was realized by a Vistec SB 350 OS e-beam writer using a lattice aperture for ultrafast writing of large areas. The advantage of the lattice aperture is that only the area, but not the period of the grating, determines the writing time. Subsequently the resist pattern was used as mask for structuring the chromium layer in an inductively coupled plasma (ICP) etching process. By means of another ICP etching process, the tempered polymer layer was structured against the chromium hard mask. The following steps are shown in Figure 1a-d. ALD films were deposited in a Beneq TFS 200 equipment. For the iridium deposition on top of the polymer grating, Ir(acac)_3 _and O_2 _were used as precursors. The metal precursor was heated to 150°C. The required process temperature for the Ir-ALD process is 350°C, exceeding the decomposition temperature of the polymer. Thus, an advance step was introduced to protect the polymer grating from decomposition, the ALD deposition of a thin (less then 5 nm) Al_2_O_3 _film as protective layer. Alumina has been deposited with a standard trimethylaluminium (TMA)/water ALD process at 150°C, thus not affecting the polymer grating.

**Figure 2:**
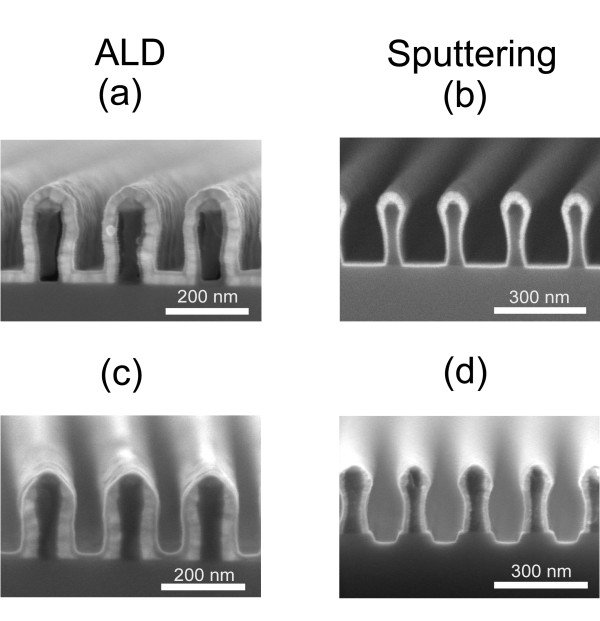
**SEM images of the fabrication process**. The overcoated polymer gratings by ALD (a) and sputter deposition (b) and the grating structures after ion beam etching (c)-(d) are shown.

**Figure 3:**
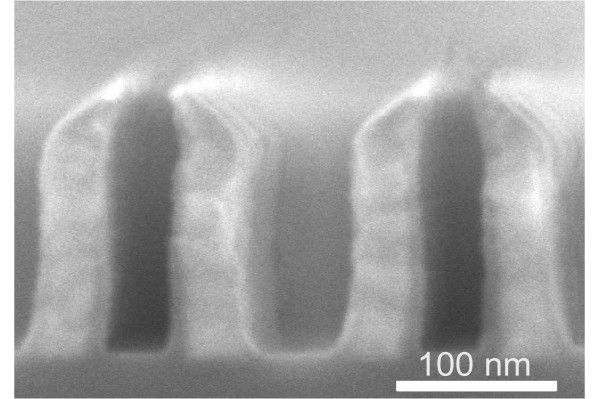
**SEM image of the 100 nm period iridium wire grid polarizer**.

Figure 2 shows the deposited metal layer obtained from sputter deposition (b) and the ALD process (a) in direct comparison. In both the cases, the nominal film thickness on the plane substrate is 30 nm. The ALD film covers the polymer grating very conformally. In contrast to the ALD film, the iridium film after sputter deposition shows poor step coverage with most of the iridium being located on top of the ridges, typical for a line-of-sight deposition. Iridium films with lower thickness are found on the sidewalls of the ridges and in the grooves. Increasing the layer thickness by means of sputter deposition will enhance the shadowing effects on the ridges and the non-conformality of the deposited film. After the deposition, the iridium located on top of the ridges and in the grooves was removed by means of ion beam etching. Figure 2c and 2d shows both resulting structures. It is obvious that the remaining iridium layer on the sidewalls of the ALD-coated polymer grating (c) is much thicker than the remaining layer on the sputter coated grating (d). In both cases the metal layer on the sidewalls increases because of the redeposited metal from the bottom of the grooves. It can be seen that ALD provides a much better control of the geometrical properties of the metal layer. Finally, the resist grating is removed by means of ICP etching so that only the metal ridges are remaining. The period of the resulting high aspect ratio grating is 100 nm. A scanning electron microscope (SEM) image of the final grating structure is shown in Figure 3.

## 2 Results and discussion

**Figure 4:**
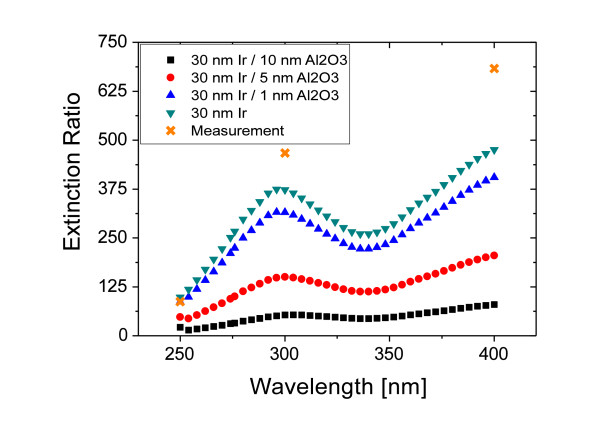
**Spectral simulation of the optical properties of the fabricated wire grid polarizer**. The influence of the protective Al_2_O_3 _layer to the extinction ratio for different layer thicknesses compared to the measured parameters is shown.

**Figure 5:**
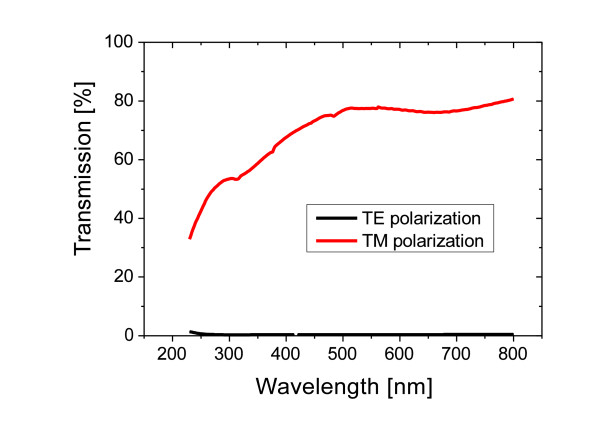
**Spectral measurement of the transmission for TE- and TM-polarized light of the fabricated polarizer**.

For the fabrication of the high-frequency iridium grating using ALD, it is necessary to protect the polymer grating from decomposition with an thin Al_2_O_3 _layer. For an accurate discussion of the optical properties of the fabricated iridium polarizer, it is important to study the influence of the protective Al_2_O_3 _layer on the transmittance and the extinction ratio. Figure 4 shows a rigorous simulation of the extinction ratio for different values of the Al_2_O_3 _layer thickness performed with the software Grating Solver [18]. The parameters used for the simulated grating are a period of 100 nm, a ridge width of 30 nm, a height of 150 nm and the values 1, 5, and 10 nm for the Al_2_O_3 _layer thickness. Increasing the thickness of the oxide layer leads to an increase in the transmission for TE- and TM-polarized light but also to a decrease in the extinction ratio. The high index alumina layer raises the effective refractive index between the metal stripes leading to an enhanced transmission [19]. Hence, the thickness of the protective Al_2_O_3 _layer should be less than 5 nm to ensure a high extinction ratio. The measurement of the spectral optical properties of the fabricated structure was taken with a Perkin Elmer Lambda 950 two-beam spectrometer. In the wavelength range from 800 to 230 nm, the spectral transmission for TE- and TM-polarized light was measured versus the wavelength. To exactly determine the extinction ratio, the transmission was measured versus the polarizer angle for selected wavelengths. The characteristic minimum and maximum value of the measured curve represents the TE- and TM polarization, respectively. This measurement should avoid alignment mistakes between the polarization direction of the incoming light and the polarizer, since minor changes in the value of the TE polarization can significantly effect the extinction ratio. Figure 5 shows a plot of the spectral transmission from the IR down to the UV spectral region for the fabricated 100 nm period iridium grating. The extinction ratio of the polarizer in the visible spectral range at a wavelength of 500 nm amounts to 889, at 400 nm to 683, in the UV range at 300 nm to 467, and at 250 nm to 87. Compared to the extinction ratio of a polarizer fabricated using sputter deposition [11], the values obtained with the ALD technology are considerably higher. For example, at 300 nm wavelength, the extinction ratio for an ALD-based polarizer is 467, while the corresponding sputter-based polarizer shows an extinction ratio of 30 only. This is caused by the limited ridge width of the sputter-based fabrication where a larger ridge width leading to a high extinction ratio could not be realized. Hence, the ALD technology is a unique and superior way to accurately adjust the grating parameters in a wide range according to the requirements of the targeted application.

## 3 Conclusions

In conclusion, we have implemented the ALD technology in a frequency doubling process for the fabrication of a high aspect ratio iridium wire grid polarizer. Besides that, we showed that ALD technology is a more suitable deposition technique for the frequency doubling process than sputter deposition. The broadband optical function of the polarizer was shown from the IR down to a wavelength of 230 nm. Furthermore, the results were compared with a polarizer fabricated using a sputter technique and showed a notably higher extinction ratio in the whole investigated spectral range. A decrease of the target wavelength below 200 nm requires smaller grating periods and is the aim of further investigations.

## Abbreviations

TM: transverse magnetic; TE: transverse electric; ALD: atomic layer deposition; ICP: inductively coupled plasma; TMA: Trimethylaluminium; SEM: scanning electron microscope

## Competing interests

The authors declare that they have no competing interests.

## Authors' contributions

TW coordinated the study, carried out the theoretical simulations as well as the optical characterization, and wrote the manuscript. TK performed the sputter deposition and etching experiments. AS and MK carried out the ALD deposition and gave helpful suggestions for the manuscript. EBK and AT provided the experimental conditions and revised the manuscript critically. All authors read and approved the final manuscript.
